# Prospective study of a case-finding algorithm to detect NAFLD with advanced fibrosis in primary care patients

**DOI:** 10.1097/HC9.0000000000000024

**Published:** 2023-02-01

**Authors:** Rena K. Fox, Janet N. Chu, Max L. Goldman, Kendall B. Islam, Danielle Brandman

**Affiliations:** 1 Division of General Internal Medicine, Department of Medicine, University of California, San Francisco, California, USA; 2 Division of Hospital Medicine, Department of Medicine, San Francisco Veterans Affairs Medical Center and University of California, San Francisco, California, USA; 3 School of Medicine, University of California, San Francisco, California, USA; 4 Division of Gastroenterology and Hepatology, Department of Medicine, Weill Cornell Medical College, New York, New York, USA

## Abstract

Primary care providers need strategies to identify NAFLD patients and select for specialty referral, but proposed algorithms have only been studied in established NAFLD patients.

**Methods:**

We implemented an algorithm for all adults with diabetes mellitus in a large primary care practice and excluded hepatitis B and C or alcohol use. Applying annual Fibrosis-4 Index and NAFLD Fibrosis Score for 5 years, we categorized patients as low-risk, indeterminate-risk, or high-risk for advanced fibrosis. We targeted all high-risk and messaged each primary care provider, recommending hepatology linkage. We collected final diagnosis and fibrosis (F0–4) outcomes. Using multivariable logistic regression, we assessed risk factors for advanced fibrosis stage (F3–4).

**Results:**

Of 3028 patients, 1018 were low-risk, 577 indeterminate-risk, and 611 high-risk. There were 264 target patients; their 89 primary care providers received a message per patient suggesting hepatology referral. The majority (n=149) were referred; at triage, 118 were deemed likely NAFLD. Of these, 90 completed visits, 78/90 were diagnosed as NAFLD, and 69/78 underwent fibrosis staging, with F3 to 4 in 25/69. In multivariable analysis, hemoglobin A1c ≥8% (OR=7.02, 95% CI: 1.29–38.18) and Fibrosis-4 Index (OR=1.79, 95% CI: 1.07–2.99) were associated with increased risk of F3 to 4.

**Conclusions:**

This is the first prospective study testing a case-finding strategy in primary care and almost 1/3 of diabetes mellitus were high-risk for advanced fibrosis. When prompted, 73% of primary care providers placed referrals and 76% of patients completed visits, revealing 86% NAFLD and 36% F3 to 4. This study demonstrates the readiness for such a strategy in primary care; integrating hemoglobin A1c into this algorithm may further improve the performance of Fibrosis-4 Index in this setting.

## Graphical abstract

**Figure:**
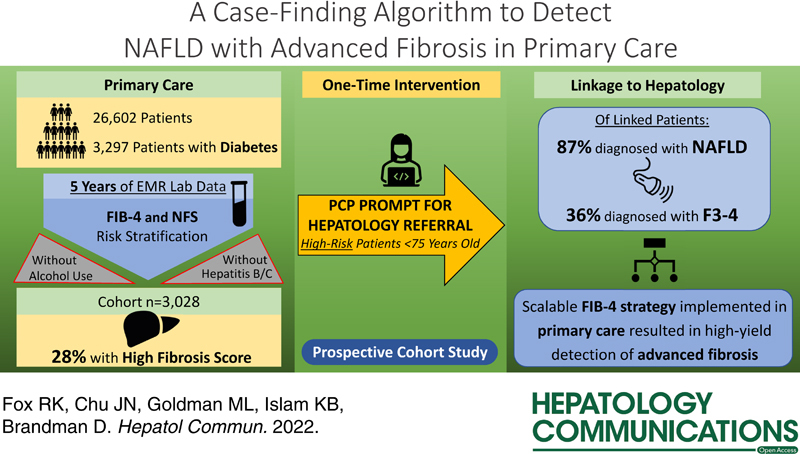

## INTRODUCTION

NAFLD encompasses a spectrum of histological features, from simple steatosis to NASH to cirrhosis.^1^ Approximately 25% to 37% of the US adult population is estimated to have NAFLD, of whom 30% have NASH.^2^–^5^ The prevalence of NAFLD and NASH are even higher among those with metabolic syndrome and diabetes mellitus (DM), with up to 70% of patients with DM having NAFLD.^6^,^7^ Individuals with NASH are at the highest risk of progression to cirrhosis and liver cancer; 20% of patients with NASH are estimated to progress to cirrhosis, while this is true in ~5% of NAFLD patients.^8^,^9^

Early detection of advanced fibrosis in NAFLD is essential, as fibrosis stage is the most important predictor of liver-related complications, including variceal hemorrhage, ascites, hepatocellular carcinoma, and liver-related mortality.^10^–^12^ Identifying patients in the primary care setting is ideal, in order to intervene early with counseling, weight loss strategies, and treatment. However, recognizing NAFLD in primary care presents an enormous challenge: patients are usually asymptomatic, there is no one straightforward diagnostic test, and no practice guidelines in the United States currently recommend screening for any subgroup of the population. Therefore, it is not surprising that 25% of NAFLD patients already have a moderate or high level of fibrosis at the time of diagnosis.^13^

In addition to lacking a standardized NAFLD diagnostic approach for the extraordinarily high number of patients in primary care with risk factors, primary care providers (PCPs) must also decide when or if to refer patients to specialty care. Given the size of the NAFLD population, referral should be used selectively and only for patients at the highest risk of disease progression. PCPs therefore need an efficient and well-validated strategy to estimate fibrosis risk to guide a decision on referral. Noninvasive testing such as transient elastography (TE), ultrasound-based elastography, and magnetic resonance elastography can detect advanced fibrosis with high correlation to liver biopsy,^14^–^16^ yet these technologies are limited by the expense and low availability. Clinical calculators such as the Fibrosis-4 Index (FIB-4) and NAFLD Fibrosis Score (NFS) are composed of common widely available blood tests, however, have been validated only in patients with an established NAFLD diagnosis within the hepatology clinic or research setting.^17^–^21^

Recently, the American Gastroenterological Association (AGA) and others published a proposed algorithm for the management of patients with NAFLD, including the use of FIB-4 to differentiate which patients would be managed by PCPs and which would be referred to hepatology specialty care.^4^,^22^ However, this strategy was not implemented or prospectively tested in a primary care setting, and the algorithm relied on an existing NAFLD diagnosis before proceeding to risk stratification by FIB-4.^22^,^23^ No study, to our knowledge, has yet tested a strategy using the FIB-4 or NFS as a first step applied directly to primary care patients at risk for NAFLD but prior to diagnosis.

We aimed to implement and prospectively evaluate the ability of a case-finding algorithm to identify NAFLD with advanced fibrosis in all primary care patients with DM and increase their access to specialty care, by first applying FIB-4 and NFS and then linking high-risk patients to hepatology for evaluation and fibrosis staging.

## METHODS

### Study design and setting

We performed a prospective study at the University of California, San Francisco (UCSF) in the General Medicine Practice (GMP), the main adult primary care practice which serves an ethnically and socioeconomically diverse urban population of 26,602 adult patients. The patient mix is 47% White, 23% Asian, 9% Hispanic/Latino, and 9% Black/African American, and the payer mix is 34% private insurance, 16% Medicare, and 10% Medi-Cal (Medicaid). Approximately 60,000 visits are conducted annually.

### Patient cohort and eligibility criteria

Using the electronic medical record (EMR) we identified all adult patients (age 18 y or above) with DM who were actively engaged in primary care. Active engagement in care was defined as having at least 1 in-person visit or patient-initiated digital message written to the PCP within the prior 3 years (July 1, 2017–June 30, 2020). DM was defined as having at least 1 of the following criteria: (1) active problem list included a DM ICD 9/10 code, (2) hemoglobin A1c (HbA1c) ≥6.5% at least once in the prior 2 years, or (3) current medication list including at least 1 antidiabetes medication (Supplemental Table 1, http://links.lww.com/HC9/A91). Patients with gestational diabetes, prediabetes, or steroid-induced diabetes were excluded.

We excluded any patient who had existing evidence of common non-NAFLD liver diseases, specifically chronic HBV, chronic HCV, or alcohol use disorder. Exclusionary criteria were defined as detectable HBV surface antigen, detectable HCV RNA, or ICD 9/10 code for alcohol use disorder (Supplemental Table 2, http://links.lww.com/HC9/A91) recorded during any time in the patient’s medical record, not limited to the study period. ICD 9/10 codes were not used for excluding chronic HBV or HCV given inaccuracy of codes alone without existing lab data. We did not differentiate HCV patients who had achieved a prior sustained virological response; patients with any prior detectable HCV RNA were excluded.

The final cohort consisted of all adult primary care patients with DM and without common competing causes of liver disease.

### Data collection

Using existing data in the EMR, we extracted demographic, radiographic, and laboratory data in addition to ICD 9/10 codes for the cohort. We identified existing evidence of radiographic steatosis with a review of all abdominal ultrasounds performed for the entire cohort over the 20 years prior to the study period (2000–2020). The full text for all associated ultrasound reports were then extracted from the EMR and using natural language processing techniques, we identified all studies positive for hepatic steatosis, selecting for terms “fatty liver” and “steatosis.”

### Computation of FIB-4 and NFS scores

FIB-4 and NFS were computed from existing lab and clinical data in the EMR: aspartate transaminase, alanine aminotransferase (ALT), platelet and age for both scores, and additionally albumin, body mass index (BMI), and presence of DM for NFS. We used outpatient data only, excluding testing from Emergency Departments or inpatient settings. We then calculated 1 FIB-4 and 1 NFS per patient per calendar year of the study period (2015–2020). To compute the most recent FIB-4 and NFS for each year, rather than the highest or an average across the year, we selected the data points closest to the end of each calendar year (eg, last aspartate transaminase of the year). Data extraction was performed in July 2020, with the assumption that the onset of the COVID-19 pandemic in March 2020 may have resulted in fewer patients having available labs for 2020. If all components were available within the calendar year, patients would have one FIB-4 and NFS calculated for that year, thus up to 6 FIB-4 and 6 NFS scores were possible per patient for the study period. If patients lacked a component for the entire calendar year, no score was computed that year. If a patient lacked the necessary data for each year of the study period, then they had no available score.

### Risk group categorization

**FIGURE 1:**
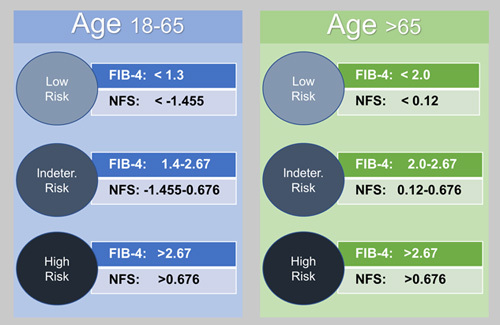
Definition of Fibrosis-4 Index (FIB-4) and NAFLD Fibrosis Score (NFS) score risk stratification using age-based cutoffs.

We selected the highest FIB-4 from the 6 possible available FIB-4s for each patient and the highest NFS by the same process. Using their highest FIB-4 and highest NFS, we categorized each patient into risk groups of low-risk, indeterminate-risk, or high-risk for advanced fibrosis, using established age-based score cutoffs for NAFLD (Figure 1). The highest FIB-4 and NFS did not have to come from the same year. For patients with discordant FIB-4 and NFS categories, we assigned a final risk group based on the algorithm provided in Supplemental Figure 1, http://links.lww.com/HC9/A81. The demographic and clinical characteristics of the patients without any FIB-4 or NFS were compared to patients with available scores (Supplemental Table 3, http://links.lww.com/HC9/A91).

### Clinical validation of NAFLD in high-risk patients

Since inclusion criteria for the cohort required the presence of DM, but did not require pre-existing diagnostic elements of NAFLD, we validated whether patients with high-risk FIB-4/NFS scores were clinically compatible with NAFLD by manual chart review. A representative sample of 10% of the high-risk group was randomly selected, and 2 investigators (D.B., R.F.) manually reviewed all electronic charts in the sample.

### Target group for intervention

The target group for the intervention of linkage to specialty care was all primary care DM patients younger than 75 years old categorized as high-risk for advanced fibrosis by FIB-4 and/or NFS. We used age as a criterion in order to provide linkage to the patients most likely to benefit from the intervention.

### Intervention: linkage to specialty care

All PCPs were notified in advance about the study processes and implementation of our algorithm. PCPs were also provided with a short educational update on NAFLD and the rationale for referral of patients with high-risk scores. A patient navigator (K.I.) sent 1 prompt within the EMR to each PCP for all patients in the target group, informing the PCP that the patient had FIB-4 and/or NFS scores that categorized them as being at high-risk for NAFLD with advanced fibrosis. The prompt also recommended that the patient be referred to hepatology for further evaluation, and a pended electronic order for hepatology referral was included. In addition, educational information on diagnosing and managing NAFLD was included for the PCP. The PCP had the option to sign the hepatology referral and to choose how to inform the patient of the referral. The study team had no contact with patients at any time. If the PCP referred, patients were added to the same pool and their referral went through the same scheduling process as all other incoming hepatology referrals, in which a hepatology clinic nurse reviewed referred patients for appropriateness. Patients were either recommended for a full consultation (in-person or video visit), TE only, or e-consult in which a hepatologist provides recommendations to the PCP but does not see the patient. Those recommended for consultation were scheduled with any of the multiple UCSF hepatology faculty, unrelated to the study. The need for and type of fibrosis assessment was determined by the hepatologist during consultation.

An electronic anonymous PCP follow-up survey was conducted after completion of the study asking about their experience with the intervention.

### Variables

The primary outcome of interest was advanced fibrosis or cirrhosis (F3–4), versus no advanced fibrosis (F0–2), defined as (1) F3 or F4 fibrosis score by TE using Fibroscan, by liver stiffness measurement >8 kPa, magnetic resonance elastography, and/or liver biopsy, and/or (2) definitive radiological evidence of hepatic cirrhosis (eg, nodular liver, signs of portal hypertension). The primary predictors of interest were age, race/ethnicity, BMI, HbA1c, and FIB-4.

Sex, race/ethnicity, insurance status, and radiographic steatosis were categorical variables. Categorical clinical variables used cutoffs of HbA1c ≥8%, HDL <40, triglycerides >150, platelets <150. BMI was categorized using Asian-specific cutoffs for Asian patients and by World Health Organization cutoffs for all other patients. FIB-4 was a continuous variable in bivariable and multivariable analyses. NFS was not included in bivariable and multivariable analyses due to collinearity between FIB-4 and NFS. FIB-4 and NFS were designated as categorical variables in analysis of their performance characteristics for identifying advanced fibrosis.

### Statistical analysis

Demographic and clinical variables were summarized using mean and SD for age and ALT, medians and interquartile ranges for FIB-4 and NFS, and proportions for categorical variables. We compared differences between risk score groups (ie, low-risk, indeterminate-risk, and high-risk score) using analysis of variance testing for continuous variables and χ^2^ tests for categorical variables, respectively.

We assessed the association between our predictor variables of interest and our primary outcome using bivariable logistic regression. Our primary predictor variables for multivariable analyses were chosen a priori based on clinical significance (sex, race/ethnicity, and BMI) as well as statistical significance from bivariable analyses (HbA1c and median FIB-4). We then assessed the association between our primary predictor variables and advanced fibrosis stage using multivariable logistic regression. We determined no statistically significant collinearity or interaction between included variables. We assessed statistical significance at *p*<0.05. Stata 16.1 (StataCorp LLC, College Station, TX) was used to analyze the data. All research was conducted in accordance with both the Declarations of Helsinki and Istanbul, all research was approved by the UCSF Institutional Review Board, IRB# 20-29942. The IRB approved a waiver of informed consent for this research, with no contact between patients and study team.

## RESULTS

### Patient characteristics and risk group categorization

**FIGURE 2:**
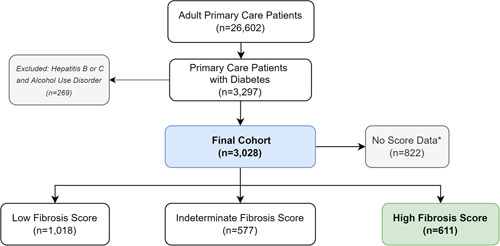
Primary care patients with diabetes classified by Fibrosis-4 Index and NAFLD Fibrosis Score.

Of 26,602 adult primary care patients, 3297 (12%) had DM. A total of 269 patients were excluded for meeting criteria for HBV, HCV, or alcohol use disorder. There were 3028 patients in the final cohort (Table 1, Fig. 2), and 2206 (73%) had existing data required to compute a FIB-4 or NFS within at least 1 calendar year. The remaining 822 (27%) patients did not have all the necessary laboratory components concurrently available within any single calendar year. Using the highest FIB-4 and NFS for each patient, 1018 (46%) were categorized as having a low-risk score, 577 (26%) an indeterminate-risk score, and 611 (28%) a high-risk score (Fig. 2). The distribution of FIB-4 and NFS scores for each risk category are shown in Table 2.

**TABLE 1:** Characteristics of primary care patients with diabetes, categorized by FIB-4 and NFS risk category

Variable	Total cohort, n (%)	FIB-4 and/or NFS available^a^, n (%)	Low-risk group, n (%)	Indeterminate-risk group, n (%)	High-risk group, n (%)	*p*
	n=3028	n=2206	n=1018	n=577	n=611	
Age [mean (SD)]	67 (14)	68 (14)	63 (15)	68 (12)	76 (11)	<0.001
Men	1470 (48.5)	1031 (46.7)	427 (41.9)	297 (51.5)	307 (50.2)	<0.001
Race/ethnicity						0.032
White	809 (27.3)	602 (27.8)	266 (26.6)	162 (28.7)	174 (28.8)	
Black	434 (14.7)	351 (16.2)	165 (16.5)	106 (18.8)	80 (13.2)	
Latinx	357 (12.1)	280 (12.9)	141 (14.1)	66 (11.7)	73 (12.1)	
Asian	1076 (36.3)	742 (34.2)	324 (32.4)	188 (33.3)	230 (38.0)	
Other	286 (9.7)	194 (8.9)	104 (10.4)	42 (7.4)	48 (7.9)	
Insurance type						<0.001
Public	1969 (65.0)	1507 (68.3)	607 (59.6)	397 (68.8)	503 (82.3)	
Private	950 (31.4)	622 (28.2)	372 (36.5)	152 (26.3)	98 (16.0)	
Uninsured	109 (3.6)	77 (3.5)	39 (3.8)	28 (4.9)	10 (1.6)	
BMI^b^						<0.001
Underweight	29 (1.0)	22 (1.0)	6 (0.6)	11 (1.9)	5 (0.8)	
Normal	425 (14.0)	347 (15.7)	152 (14.9)	82 (14.2)	113 (18.5)	
Overweight	656 (21.7)	523 (23.7)	219 (21.5)	133 (23.1)	171 (28.0)	
Obese	1918 (63.3)	1314 (59.6)	641 (63.0)	351 (60.8)	322 (52.7)	
Hypertension ICD code	2494 (82.4)	1864 (84.5)	799 (78.5)	500 (86.7)	565 (92.5)	<0.001
Hyperlipidemia ICD code	2463 (81.3)	1811 (82.1)	795 (78.1)	482 (83.5)	534 (87.4)	<0.001
NASH/NAFLD ICD code	341 (11.3)	302 (13.7)	130 (12.8)	80 (13.9)	92 (15.1)	0.43
Cirrhosis ICD code	64 (2.1)	63 (2.9)	6 (0.6)	12 (2.1)	45 (7.4)	<0.001
Median FIB-4 (IQR)^c^	1.4 (1.0–2.0)	1.4 (0.99–1.96)	1.0 (0.8–1.3)	1.5 (1.3–1.8)	2.3 (1.9–2.8)	<0.001
Median NFS (IQR)^d^	0.2 (−0.8 to 1.0)	0.17 (−0.80 to 0.97)	−0.9 (−1.5 to −0.2)	0.2 (−0.4 to 0.7)	1.2 (0.8–1.7)	<0.001
Hemoglobin h ≥8	790 (26.1)	527 (23.9)	265 (26.0)	138 (23.9)	124 (20.3)	0.032
ALT [mean (SD)]	26 (14)	26 (14)	26 (14)	26 (14)	25 (14)	0.12
Platelet <150	309 (10.2)	279 (12.6)	7 (0.7)	46 (8.0)	226 (37.0)	<0.001
HDL <40	742 (24.5)	1652 (74.9)	225 (22.1)	168 (29.1)	161 (26.4)	0.007
Triglyceride ≥150	1170 (38.6)	850 (38.5)	414 (40.7)	217 (37.6)	219 (35.8)	0.13
Hepatology visit prior	121 (4.0)	117 (5.3)	33 (3.2)	37 (6.4)	47 (7.7)	<0.001
Abdominal imaging (ever)	1792 (59.2)	1515 (68.7)	611 (60.0)	408 (70.7)	496 (81.2)	<0.001
Steatosis by ultrasound	n=1206	n=1045	n=403	n=277	n=365	
	654 (54.2)	574 (54.9)	242 (60.0)	145 (52.3)	187 (51.2)	0.030

^a^ Eight hundred and twenty-two patients did not have available data to calculate a risk score and therefore could not be assigned to a risk group based on our risk group assignment strategy.

^b^ BMI categories using different cutoffs for Asians and non-Asians: underweight=BMI <18.5 for both Asians and non-Asians; normal=BMI 18.5 to 24.9 for non-Asians and BMI 18.5 to 22.9 for Asians; overweight=BMI 25 to 29.9 for non-Asians and 23 to 26.9 for Asians; obese=BMI ≥30 for non-Asians and BMI ≥27 for Asians.

^c^ Calculated by taking the median of the highest FIB-4 for each patient.

^d^ Calculated by taking the median of the highest NFS for each patient.

Abbreviations: ALT, alanine aminotransferase; BMI, body mass index; FIB-4, Fibrosis-4 Index; HDL, high-density lipoprotein; IQR, interquartile range; NFS, NAFLD Fibrosis Score.

**TABLE 2:** Distribution of patient FIB-4 and NFS scores, categorized by risk of advanced fibrosis

	FIB-4 (n)^a^			
NFS (n)^a^	Low	Indeterminate	High	Total [n (%)]
Low	327	21	4	352 (16.0)
Indeterminate	380	250	19	649 (29.4)
High	161	285	249	695 (31.5)
Missing NFS data	311	146	53	510 (23.1)
Total [n (%)]	1179 (53.5)	702 (32.8)	325 (14.7)	2206

^a^ Refer to Figure 1 for definition of FIB-4 and NFS score risk stratification.

Abbreviations: FIB-4, Fibrosis-4 Index; NFS, NAFLD Fibrosis Score.

Table 1 demonstrates the baseline characteristics of the total cohort and compares patients by risk groups. The mean age was 67 years old (SD: 14), and those in the high-risk group were older than those in the low-risk or indeterminate-risk groups (*p*<0.001). About half of all patients were men. Over one-third (36%) identified as Asian-American, 27% White, 15% Black American, and 12% Latinx. A majority had public insurance (65%), and a greater proportion of those in the high-risk group had public insurance compared to the other risk groups (*p*<0.001). Over half of patients were obese (63%); those in the low-risk group were more likely to be obese compared to those in the high-risk group (*p*<0.001).

The majority of patients in the final cohort had a history of hypertension (n=2494; 82%) and hyperlipidemia (n=2463; 81%), with a higher percentage among those in the high-risk group compared to those in the low-risk group. A majority of the cohort had a HbA1c <8% (74%). Almost all patients (98%) had at least one elevated ALT between 2015 and 2020 (ALT≥19 for women, ALT ≥30 IU/L for men), yet the mean ALT for the cohort was in the normal range overall and across all risk score groups. Our validation with manual chart reviews of a random 10% of the high-risk group revealed 53% of patients were clinically consistent with NAFLD, yet only 11% (n=341) had previous ICD codes for NAFLD or NASH, and this was not significantly different among the three risk groups (*p*=0.43).

### Outcomes of PCP messaging and linkage to care

**FIGURE 3:**
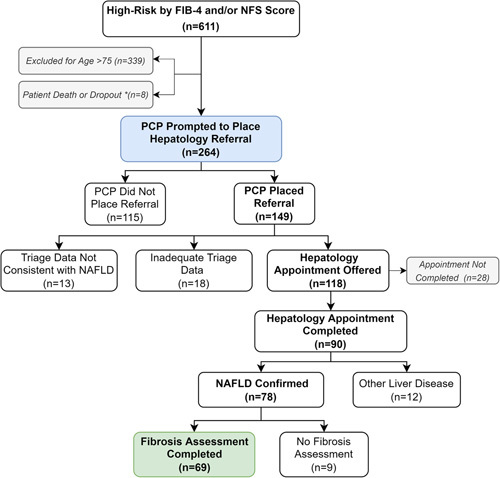
Hepatology referral pathway. Abbreviations: FIB-4, Fibrosis-4 Index; NSF, NAFLD Fibrosis Score; PCP, primary care provider.

Figure 3 illustrates the implementation of the linkage-to-care intervention for the target group. Of the 611 patients in the high-risk group, 339 were excluded for age above 75, and 8 patients were excluded for death or departure from the healthcare system before the time of the intervention. The target group therefore consisted of 264 patients. Each PCP for these target patients was sent an EMR message with the recommendation that the patient be referred to hepatology. Over half of the patients in the target group (n=149; 56%) were referred to hepatology. There were no statistically significant differences based on age, sex, race/ethnicity, insurance type, BMI, or median FIB-4 score between patients who were and were not referred to hepatology (Supplemental Table 4, http://links.lww.com/HC9/A91).

Of the patients who were referred, most (n=118; 79%) were deemed to be appropriate for hepatology consultation and likely to have NAFLD when triaged by the hepatology clinic using the routine processes for any referred patients. These patients were therefore contacted to schedule an appointment. Previsit data for 13 patients were not consistent with NAFLD, and therefore a hepatology visit was not scheduled. In addition, at triage, 18 patients were determined to have inadequate previsit data and were recommended to complete additional testing (eg, ultrasound) before the end of the study period. Of the 118 patients who were referred and offered appointments with hepatology, 90 patients (76%) completed their hepatology visit.

### PCP response to intervention

There were 147 unique PCPs who had at least one patient in the cohort, averaging 20.6 patients per PCP. Of these, 89 PCPs had at least 1 patient in the target group and received the message recommending hepatology referral, averaging 3 patients per PCP. Sixty-five of the 89 PCPs who were messaged (73%) chose to refer at least 1 of their patients. A minority of the PCPs (n=32; 36%), opted to refer all of their patients who were eligible for referral. Our follow-up survey had 68/119 respondents (57%), and the PCPs who chose not to refer 1 or more patients answered multiple-choice options for their reasons for not referring (multiple answers were allowed): 34% of PCPs reported that there were other contemporaneous competing health priorities for that patient and 20% reported that the patient was offered referral, but the patient declined or could not be reached. Other reasons included the impact of COVID and a lack of PCP confidence on the topic. Additionally, 97% of the PCP respondents reported that receiving the notification about a high-risk patient was somewhat or very useful. Furthermore, 70% reported that it helped them recognize cases of NAFLD of which they were not aware, and 47% reported that it taught them to use the FIB-4.

### Detection of NAFLD with advanced fibrosis

**FIGURE 4:**
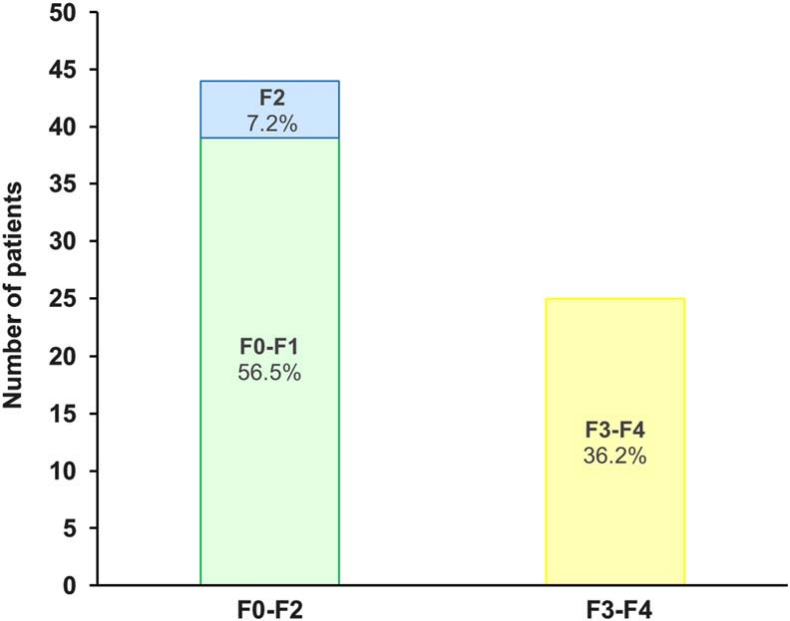
Fibrosis stage results of algorithm-identified primary care patients with diabetes.

Of the 90 patients who completed hepatology visits, 78 (87%) were diagnosed with NAFLD. Fibrosis staging was completed by 69 of these patients; 51 (74%) were staged by TE, 6 (9%) by magnetic resonance elastography, 5 (7%) by ultrasound elastography, 2 (3%) by biopsy, and 5 (7%) using relevant clinical information. Of the 69 patients who completed staging evaluations, 25 (36%) identified through our algorithm were confirmed to have advanced fibrosis or cirrhosis due to NAFLD, with the remainder having stage 0 to 2 fibrosis (Fig. 4).

### Performance characteristics of FIB-4 and NFS for advanced fibrosis in primary care

Table 3 shows the sensitivity, specificity, negative predictive value, and positive predictive value of FIB-4, NFS, and the combination of FIB-4 and NFS in identifying advanced fibrosis due to NAFLD. The sensitivity and specificity of FIB-4 was 60% and 59%, respectively. The sensitivity of NFS was higher (96%) but with much lower specificity (7%) relative to FIB-4 in our study. The overall sensitivity of combining FIB-4 and NFS as a part of our algorithm was 56%, with a specificity of 66%.

**TABLE 3:** Performance characteristics of high FIB-4 and NFS^a^ to identify advanced fibrosis in patients with NAFLD

Score	Patients with high scores among those with fibrosis staging (n/n)	Sensitivity^b^ [% (n)]	Specificity^b^ [% (n)]	PPV^b^ [% (n)]	NPV^b^ [% (n)]
High FIB-4	33/69	60.0 (15/25)	59.0 (26/44)	45.5 (15/33)	72.2 (26/36)
High NFS	65/69	96.0 (24/25)	6.8 (3/44)	36.9 (24/65)	75.0 (3/4)
High FIB-4 and high NFS	29/69	56.0 (14/25)	65.9 (26/44)	48.3 (14/29)	72.5 (29/40)

^a^ For patients with data available to compute FIB-4 and NFS, the highest FIB-4 and NFS score for each patient were used to categorize each patient as low-, indeterminate-, or high-risk for advanced fibrosis, using established cutoffs for NAFLD. We determined performance characteristics for high FIB-4, high NFS, or both in identifying advanced fibrosis in patients with NAFLD.

^b^ Sensitivity=patients with a high-risk score/patients with advanced fibrosis (F3–4); specificity=patients who do not have a high-risk score/patients without advanced fibrosis (F0–2); PPV=patients with advanced fibrosis (F3–4)/patients with a high-risk score; NPV=patients without advanced fibrosis (F0–2)/patients who do not have a high-risk score.

Abbreviations: FIB-4, Fibrosis-4 Index; NFS, NAFLD Fibrosis Score.

### Bivariable and multivariable predictors of advanced fibrosis

In bivariable analyses (Table 4), the median FIB-4 (OR=1.47; 95% CI: 1.01–2.15) and poorly controlled DM (HbA1c ≥8%) (OR=3.89; 95% CI: 1.01–14.98) were both independently and significantly associated with advanced fibrosis. In multivariable analyses controlling for sex, race/ethnicity, and BMI, those with poorly controlled DM (HbA1c ≥8%) had higher odds of having advanced fibrosis (OR=7.02; 95% CI: 1.29–38.18) compared to those with well-controlled DM. For each unit increase in median FIB-4, odds of advanced fibrosis increased 1.79 times (OR=1.79; 95% CI: 1.07–2.99). Sex, race/ethnicity, and BMI were not statistically significantly associated with advanced fibrosis.

**TABLE 4:** Bivariable and multivariable predictors of advanced fibrosis among primary care patients with NAFLD

Variable	Bivariable analysis OR (95% CI)	Multivariable analysis OR (95% CI)
Age	0.94 (0.87–1.01)	
Men (ref. women)	0.43 (0.15–1.20)	0.38 (0.11–1.37)
Race/ethnicity (ref. White)		
Black or African American	0.44 (0.08–2.53)	0.43 (0.06–3.12)
Hispanic or Latinx	1.21 (0.27–5.38)	0.94 (0.16–5.45)
Asian	3.12 (0.83–11.72)	3.61 (0.75–17.33)
Other	7.29 (0.62–82.62)	2.95 (0.17–50.29)
Insurance (ref. public)		
Private	2.57 (0.80–8.31)	
Uninsured	2.25 (0.13–38.27)	
BMI (ref. normal)		
Overweight	0.96 (0.14–6.67)	2.24 (0.14–34.70)
Obesity	1.63 (0.28–9.41)	9.84 (0.62–156.07)
Hypertension	0.38 (0.08–1.88)	
HbA1c ≥8%	**3.89** (**1.01**–**14.98)**	**7.02** (**1.29**–**38.18)**
Median FIB-4	**1.47** (**1.01**–**2.15)**	**1.79** (**1.07**–**2.99)**
Hypertriglyceridemia (ref. <150)	1.56 (0.58–4.20)	
Steatosis on ultrasound or CT	1.38 (0.37–5.11)	

*Note:* Multivariable model included sex, race/ethnicity, BMI, DM control, and median FIB-4 in the analysis.

Bolded values denote statistical significance at *p*<0.05.

## DISCUSSION

In this prospective study, we implemented a straightforward case-finding algorithm designed to detect NAFLD with advanced fibrosis in primary care patients with DM and found over two-thirds of patients had indeterminate-risk or high-risk FIB-4 and/or NFS scores. Among the high-risk patients then linked to hepatology, 87% were confirmed to have NAFLD, and 36% had advanced fibrosis, indicating the ability of our algorithm to detect advanced fibrosis in a substantial portion of patients who might otherwise have gone unrecognized. Independent risk factors for advanced fibrosis included FIB-4 and poorly controlled DM, defined as HbA1c ≥8%.

This study is unique in that all previous variations of a FIB-4 strategy for risk stratification have been limited to hepatology cohorts,^24^,^25^ introducing selection bias with regards to the providers who are specialists and the patients who are already aware of their NAFLD diagnosis. Some published expert recommendations have promoted such algorithms for primary care but only for patients with existing ultrasound-positive steatosis or elevated transaminases.^4^ The current study is the first to demonstrate an effective “FIB-4 first” strategy for detecting advanced fibrosis in a primary care setting without first selecting patients who meet NAFLD criteria.^23^,^26^

Our algorithm was successfully implemented within a large, diverse, multipayer system, which serves an expansive region, and the results demonstrate its ability to be adapted and easily scaled within large healthcare systems with EMRs. Seventy-three percent of patients had the laboratory data needed to compute a minimum of 1 FIB-4 within the last 5 calendar years, revealing that only a small minority of DM patients in primary care would need additional laboratory data in order to benefit from the current algorithm. Such an approach would reduce the reliance on additional resource-intensive testing such as TE without initial risk stratification. Unlike other proposed algorithms, we used only 1 minimal intervention, consisting of a singular EMR PCP prompt, with an optional electronic order identical to that used for any patient referred to hepatology through standard care. The patients’ process of scheduling appointments with hepatology clinic was also routine; patients referred by our algorithm were in the same pools as all other referred patients, highlighting the ease of integrating our algorithm into current practices. Since efforts to improve outcomes in healthcare can be labor-intensive and costly to develop, our intent was to create and test an algorithm for a real-world setting that would be easily scalable and low maintenance once established. We utilized a patient navigator to send PCPs messages and pend referrals; however, all of these steps can be accomplished by EMR programming, which would be an upfront cost for institutions but would avoid the need for such people-intensive roles. Automated clinical reminder notifications would notify PCPs of a patient with both DM and a high FIB-4 and recommend referral to hepatology in accordance with our tested algorithm. Given the ease of using the EMR as a tool for automatic data processing and reflex clinical reminders, we believe this algorithm, once integrated into a healthcare system, will be sustainable.

Our study highlighted the pervasive under-recognition of patients with NAFLD, despite the concurrence of significant known risk factors, in a clinical setting that lacks consensus guidelines for screening. Among the 2206 patients with a FIB-4 or NFS, 49% had a previous abdominal ultrasound and among these, 55% had evidence of steatosis, consistent with previously reported prevalence of NAFLD in patients with DM.^27^ Despite these already available radiology data, only 11% of patients had been identified by coding as having NAFLD, whereas obesity, hypertension, and hyperlipidemia were coded in 65% to 83% of patients. These findings emphasize the critical need for additional tools to help PCPs identify NAFLD in their at-risk patients. A reliance on elevated liver enzymes as the major screening strategy in the primary care setting is inappropriate, as this will not dependably identify patients with advanced fibrosis or cirrhosis.^28^ In addition, while liver biopsy has historically been the gold standard for diagnosis and staging, its use in wide-scale screening would be impractical due to expense, risk of complications, sampling error, and inter-rater and intrarater reliability. Use of TE in triage algorithms is appealing due to its noninvasive technique and lower cost,^29^ but its widespread use is limited by a dearth of availability. Rather, leveraging risk-prediction algorithms that use easily accessible, existing data from the EMR is a practical strategy to discern which patients are at high-risk of fibrosis from NAFLD and would benefit from advanced testing or need specialty referral, without undue burden or cost to the healthcare system, patients, or providers.

Our primary outcome was detection of advanced fibrosis and therefore we did not incorporate referral of low-risk and indeterminate-risk patients. Thus, we did not collect fibrosis testing for the entire spectrum of FIB-4 and NFS scores and our sensitivity and specificity findings may not be comparable to studies characterizing the performance of the full range of these calculators. However, targeting patients with a high-risk FIB-4 or NFS, we observed a reasonably strong performance of FIB-4 for detecting advanced fibrosis, with test characteristics indicating 60% sensitivity and 59% specificity. Interestingly, the sensitivity is in contrast to the performance of FIB-4 in patients with established NAFLD in a specialty setting, with previously reported 80% sensitivity and 56% specificity.^19^ Understanding the performance of FIB-4 in an undifferentiated primary care setting is an essential step before implementing it widely as a screening modality. Notably, none of the patients with advanced fibrosis had a low-risk FIB-4 score, although this is likely because only the high-risk group received a recommendation for hepatology evaluation. Addressing the utility of the NFS in our algorithm, we observed that many patients were categorized into the high-risk group due to a high NFS, yet for detecting advanced fibrosis, we found NFS had poor performance with 7% specificity and 37% PPV. Based on these results, we conclude that in an algorithm designed to help PCPs risk stratify NAFLD patients with advanced fibrosis, NFS would add a high number of false positives and would not be useful. In contrast, we found that poorly controlled DM and FIB-4 were both independently significantly associated with advanced fibrosis in multivariable analysis. This suggests that the addition of a HbA1c cutoff of ≥8% to the current algorithm may help further refine the selection of high-risk patients while preserving access to specialists without additional cost or undue burden on patients and providers, many of whom may have only limited access to TE or other measures of fibrosis stage.

We saw <100% yield at each step in the pathway between EMR messaging and completion of specialty workup, similar to the process in all cascades of care. Nonetheless, there were very high participation rates among the PCPs and a high case-detection rate among the patients. Of the PCPs who received notification of a high-risk patient, 73% referred at least 1 patient, and yet they were also discriminating; the majority chose to refer some of their patients, but not all. Allowing PCPs the option to refer was also successful in that only 8% (n=13) of referred patients did not appear consistent with NAFLD during usual hepatology triage. This extremely low rate of non-NAFLD among the referred patients indicates that the PCPs selected patients appropriately and reduced many non-NAFLD referrals, such as those with high FIB-4 or NFS for nonhepatic reasons. Conversely, patients who might have NAFLD, and would have benefitted from the hepatology evaluation, may not have been referred by their PCP for a wide variety of potential reasons. Respondents to our follow-up PCP survey provided a spectrum of reasons they did not refer patients, including their own low confidence on the NAFLD topic, the patient declining the referral, or the competing social and economic priorities of the patient.

In addition to testing the algorithm’s ability to detect advanced fibrosis, we had four other large goals for this study: to increase PCPs’ recognition of NAFLD in their patients with DM; to remind and teach PCPs to integrate FIB-4 into their management of NAFLD; to prompt PCPs to consider referral of high-risk NAFLD patients to specialty care; and to demonstrate to PCPs that patients with low FIB-4 can be managed in primary care and help avoid overuse of limited resources. Based on our follow-up survey, 97% of PCP respondents reported that receiving the EMR notification was very useful or somewhat useful. Furthermore, 70% reported that it helped them recognize cases of NAFLD of which they were not previously aware. Finally, from this singular intervention, 47% reported that it taught them how to properly use the FIB-4.

Our study had several limitations. First, the algorithm was not designed to screen for NAFLD or to diagnose NAFLD itself. Second, although the vast majority of patients had existing data to compute a FIB-4 and/or NFS within a calendar year, for the 27% who did not, many would have data to compute scores if we combined labs across calendar years. Third, we targeted only high-risk patients for referral to hepatology, yet patients with indeterminate scores may also benefit, and future studies should address the performance of this algorithm in the indeterminate group. Fourth, we did not have liver biopsy data on all patients, which may limit the precision of fibrosis stage assessment. Fifth, the use of 8 kPa as the liver stiffness measurement cutoff for advanced fibrosis may lead to an overestimation of advanced fibrosis prevalence, as this cutoff has an excellent negative predictive value but poor positive predictive value.^30^ Because all but 2 patients had liver stiffness measurement >10, we expect that the rate of misclassification of advanced fibrosis is relatively low. Finally, this study was conducted in 1 very large primary care practice, with socioeconomic and demographic diversity, but it was a single center, which could limit the generalizability of our findings.

In summary, given the enormous and rising burden of obesity and DM, in combination with the current and projected prevalence of NAFLD, PCPs need to be able to efficiently recognize at-risk patients and intervene in order to stave off advanced disease progression. While the majority of NAFLD patients will have mild disease and will be at low-risk indefinitely, PCPs should have better tools with which to identify and manage these patients within primary care, while appropriately selecting high-risk patients for specialty referral, given the relatively limited access to hepatologists and advanced diagnostic modalities. By leveraging widely available existing data, the FIB-4 could be an efficient strategy without adding excessive and expensive testing, such as ultrasound, for all patients with DM. The proposed algorithm can be further tailored for the primary care population by incorporating markedly elevated HbA1c to most accurately identify patients with advanced fibrosis or cirrhosis. Based on our findings, we would not recommend including the NFS in a future primary care algorithm. Rather, building automated clinical reminders applying FIB-4 to primary care patients with DM will assist PCPs in risk-stratifying and identifying NAFLD in patients who have not been previously diagnosed and who otherwise may not have fit a high-risk phenotype, with the goal of reducing liver-related morbidity and mortality.

## Supplementary Material
