# Supplementary figures and images for: Prospective study of a case-finding algorithm to detect NAFLD with advanced fibrosis in primary care patients
Source: Hepatol Commun. 2023 Feb 1;7(2):e0024. doi: 10.1097/HC9.0000000000000024 (PMC9894348; doi:10.1097/HC9.0000000000000024)

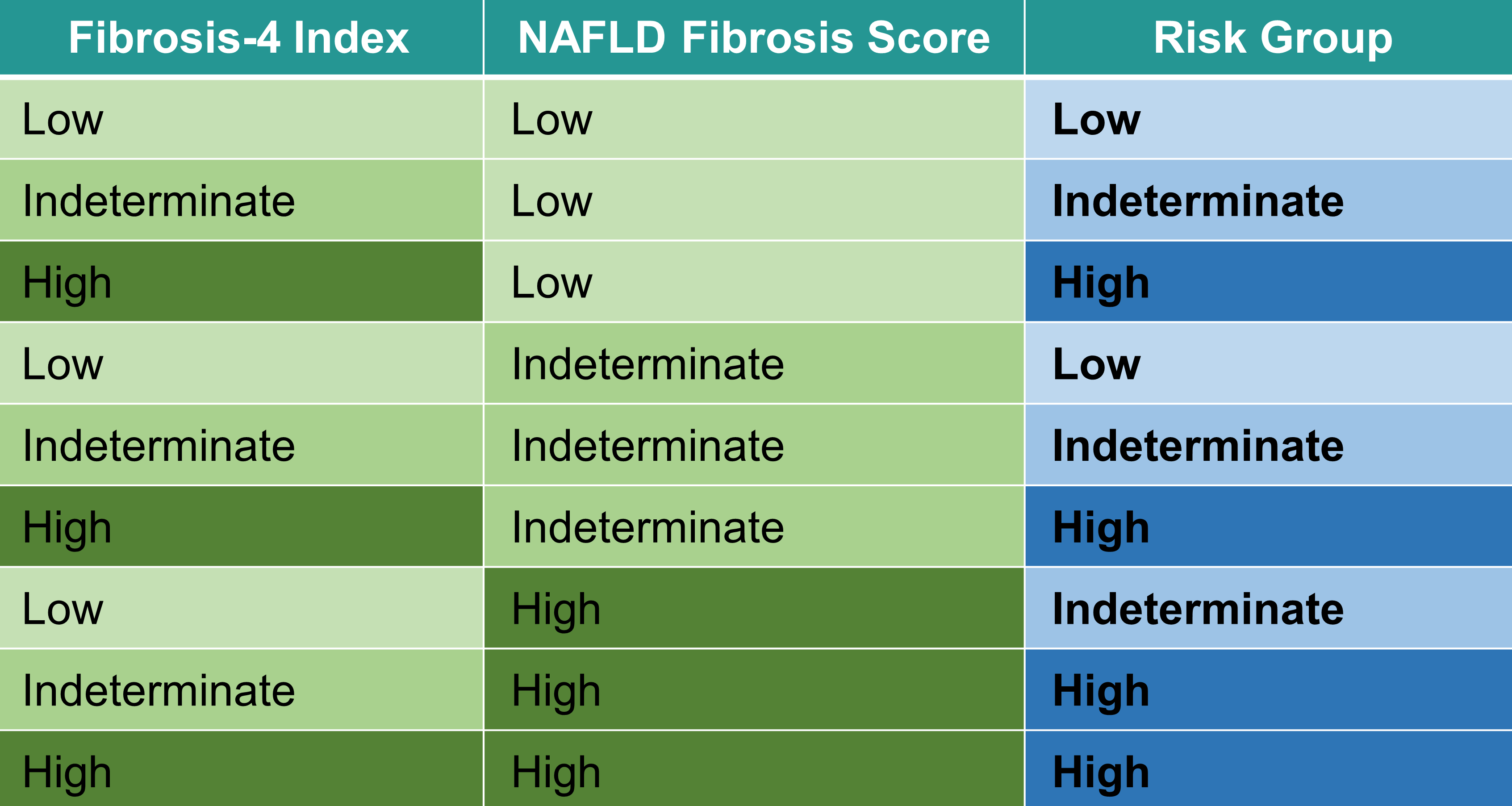

Supplement: Supplementary file 1 [file hc9-7-e0024-s001.tif]
