# Supplementary material for: Prospective study of a case-finding algorithm to detect NAFLD with advanced fibrosis in primary care patients
Source: Hepatol Commun. 2023 Feb 1;7(2):e0024. doi: 10.1097/HC9.0000000000000024 (PMC9894348; doi:10.1097/HC9.0000000000000024)
Supplement: Supplementary file 2 [file hc9-7-e0024-s002.docx]

**Supplemental Table 1.** Inclusion and Exclusion Criteria for Identification of Primary Care Patients with Diabetes Mellitus,

| **Criteria** | **Name** | **ICD 9** | **ICD 10** |
| --- | --- | --- | --- |
| **HbA1c ≥ 6.5 within the last 2 years** | “Hemoglobin A1c”  “POCT Hemoglobin A1C” |  |  |
| **Active problem list diagnosis - Inclusions** |  | '250', '357.2', '362.01', '362.02', '362.03', '362.04', '362.05', '362.06', '362.07', '366.41', '648.00', '648.01', '648.02', '648.03', '648.04' | ‘E10', 'E11', 'E13' |
| **Active med list medication** | 'ACARBOSE', 'ACTOPLUS', 'ACTOS', 'AMARYL', 'APIDRA', 'AVANDAMET', 'AVANDARYL', 'AVANDIA', 'BYDUREON', 'BYETTA', 'CHLORPROPAMIDE', 'DIABETA', 'DUETACT', 'FARXIGA', 'GLIMEPIRIDE', 'GLIPIZIDE', 'GLUCOTROL', 'GLUCOVANCE', 'GLYBURIDE', 'GLYNASE', 'GLYSET', 'HUMALOG', 'HUMULIN', 'INVOKAMET', 'INVOKANA', 'JANUMET', 'JANUVIA', 'JARDIANCE', ‘JENTADUETO', 'JUVISYNC', 'KAZANO', 'KOMBIGLYZE', 'LANTUS', 'LEVEMIR', 'LISPRO', 'METAGLIP', 'METFORMIN', 'NATEGLINIDE', 'NESINA', 'NOVOLIN', 'NOVOLOG', 'ONGLYZA', 'OSENI', 'PIOGLITAZONE', 'PRANDIMET', 'PRANDIN', 'PRECOSE', 'RELION', 'REPAGLINIDE', 'STARLIX', 'SYMLIN', 'TANZEUM', 'TOLAZAMIDE', 'TOLBUTAMIDE', 'TRADJENTA', 'VICTOZA' |  |  |

**Supplemental Table 2.** Criteria for Identification of Primary Care Patients with Hepatitis B, Hepatitis C, and Alcohol Use Disorder

| **Comorbidity** | **ICD9** | **ICD10** | **Lab data** |
| --- | --- | --- | --- |
| **Chronic hepatitis C** |  |  | Hepatitis C virus RNA detectable or quantifiable |
| **Chronic hepatitis B** |  |  | Hepatitis B virus surface antigen positive |
| **Alcohol use disorder** | 291.1, 291.2, 291.5-291.9,  303.9, 305.0, V113  291.0-291.3, 291.5, 291.8,  291.9, 303.x, 305.0  265.2, 291.1-291.3,  291.5-291.9, 303.0,  303.9, 305.0, 357.5,  425.5, 535.3,  571.0-571.3, 980.x,  V11.3 | F10, E52, G62.1, I42.6,  K29.2, K70.0, K70.3,  K70.9, T51.x, Z50.2,  Z71.4, Z72.1 |  |

**Supplemental Table 3.** Characteristics of Primary Care Patients with Diabetes, with vs. without Previous FIB-4 or NFS Scores

| **Variable** | | **Data available to calculate FIB-4 or NFS** | **Data *not* available to calculate FIB-4 or NFS** | ***P* value** |
| --- | --- | --- | --- | --- |
|  | | **n=2,206** | **n=822** |  |
| **Age [mean (SD)]** | | 68 (14) | 65 (15) | <0.001 |
| **Men** | | 1,031 (46.7%) | 439 (53.4%) | 0.001 |
| **Race/Ethnicity** | |  |  | <0.001 |
| White | | 602 (27.8%) | 207 (26.1%) |  |
| Black | | 351 (16.2%) | 83 (10.5%) |  |
| Hispanic or Latino | | 280 (12.9%) | 77 ( 9.7%) |  |
| Asian | | 742 (34.2%) | 334 (42.1%) |  |
| Other | | 194 ( 8.9%) | 92 (11.6%) |  |
| **Insurance Type** | |  |  | <0.001 |
| Public | | 1,507 (68.3%) | 462 (56.2%) |  |
| Private | | 622 (28.2%) | 328 (39.9%) |  |
| Uninsured | | 77 ( 3.5%) | 32 ( 3.9%) |  |
| **BMI** † | |  |  | <0.001 |
| Underweight | | 22 ( 1.0%) | 7 ( 0.9%) |  |
| Normal | | 347 (15.7%) | 78 ( 9.5%) |  |
| Overweight | | 523 (23.7%) | 133 (16.2%) |  |
| Obese | | 1,314 (59.6%) | 604 (73.5%) |  |
| **Hypertension** | | 1,864 (84.5%) | 630 (76.6%) | <0.001 |
| **Hyperlipidemia** | | 1811 (82.1%) | 652 (79.3%) | 0.081 |
| **NASH/NAFLD ICD code** | | 302 (13.7%) | 39 ( 4.7%) | <0.001 |
| **Abdominal imaging (ever)** | | 1,515 (68.7%) | 277 (33.7%) | <0.001 |
| **Steatosis by Ultrasound** | | n=1,045 | n=161 |  |
|  |  | 574 (54.9%) | 80 (49.7%) | 0.214 |
|  |  |  |  |  |

† BMI categories using different cutoffs for Asians and non-Asians: Underweight = BMI <18.5 for both Asians and non-Asians; Normal = BMI 18.5-24.9 for non-Asians and BMI 18.5-22.9 for Asians; Overweight = BMI 25-29.9 for non-Asians and 23-26.9 for Asians; Obese = BMI ≥30 for non-Asians and BMI ≥ 27 for Asians

**Supplemental Table 4.** Characteristics of High-Risk Primary Care Patients, Referred vs. Not Referred to Hepatology

| **Variable** | **Total** | **Referred** | **Not Referred** | ***P* value** |  |  |
| --- | --- | --- | --- | --- | --- | --- |
|  | n=262 | n=148 | n=114 |  |  |  |
| **Age [mean (SD)]** | 65 (7.6) | 65 (7.2) | 66 (8) | 0.42 |  |  |
| **Men** | 120 (45.8%) | 62 (41.9%) | 58 (50.9%) | 0.15 |  |  |
| **Race/Ethnicity** |  |  |  | 0.14 |  |  |
| White or Caucasian | 92 (35.5%) | 46 (31.5%) | 46 (40.7%) |  |  |  |
| Black or African American | 44 (17.0%) | 28 (19.2%) | 16 (14.2%) |  |  |  |
| Hispanic or Latino | 32 (12.4%) | 23 (15.8%) | 9 ( 8.0%) |  |  |  |
| Asian | 60 (23.2%) | 30 (20.5%) | 30 (26.5%) |  |  |  |
| Other | 31 (12.0%) | 19 (13.0%) | 12 (10.6%) |  |  |  |
| **Insurance Type** |  |  |  | 0.90 |  |  |
| Public | 194 (74.0%) | 108 (73.0%) | 86 (75.4%) |  |  |  |
| Private | 61 (23.3%) | 36 (24.3%) | 25 (21.9%) |  |  |  |
| Uninsured | 7 ( 2.7%) | 4 ( 2.7%) | 3 ( 2.6%) |  |  |  |
| **BMI†** |  |  |  | 0.95 |  |  |
| Underweight | 2 ( 0.8%) | 1 ( 0.7%) | 1 ( 0.9%) |  |  |  |
| Normal | 29 (11.1%) | 15 (10.1%) | 14 (12.3%) |  |  |  |
| Overweight | 64 (24.4%) | 36 (24.3%) | 28 (24.6%) |  |  |  |
| Obese | 167 (63.7%) | 96 (64.9%) | 71 (62.3%) |  |  |  |
| **Hypertension** | 232 (88.5%) | 133 (89.9%) | 99 (86.8%) | 0.45 |  |  |
| **Hyperlipidemia** | 226 (86.3%) | 134 (90.5%) | 92 (80.7%) | 0.022 |  |  |
| **NASH/NAFLD ICD code** | 58 (22.1%) | 44 (29.7%) | 14 (12.3%) | <0.001 |  |  |
| **Cirrhosis ICD code** | 31 (11.8%) | 28 (18.9%) | 3 ( 2.6%) | <0.001 |  |  |
| **Median FIB-4 (IQR)‡** | 1.98 (1.46-2.41) | 1.95 (1.41-2.40) | 2.01 (1.50-2.42) | 0.45 |  |  |
| **Median NFS (IQR)^¶^** | 0.97 (0.61-1.51) | 0.97 (0.63-1.54) | 0.97 (0.58-1.48) | 0.94 |  |  |
| **HbA1c ≥8%** | 65 (24.8%) | 32 (21.6%) | 33 (28.9%) | 0.17 |  |  |
| **ALT [mean (SD)]** | 28.0 (15.9) | 30.4 (16.7) | 24.9 (14.3) | 0.005 |  |  |
| **Platelet <150** | 100 (38.2%) | 51 (34.5%) | 49 (43.0%) | 0.16 |  |  |
| **HDL <40** | 81 (30.9%) | 44 (29.7%) | 37 (32.5%) | 0.64 |  |  |
| **TG ≥150** | 113 (43.1%) | 64 (43.2%) | 49 (43.0%) | 0.97 |  |  |
| **Hepatology visit prior** | 29 (11.1%) | 27 (18.2%) | 2 (1.8%) | <0.001 |  |  |
| **Abdominal imaging (ever)** | 218 (83.2%) | 124 (83.8%) | 94 (82.5%) | 0.78 |  |  |

† BMI categories using different cutoffs for Asians and non-Asians: Underweight = BMI <18.5 for both Asians and non-Asians; Normal = BMI 18.5-24.9 for non-Asians and BMI 18.5-22.9 for Asians; Overweight = BMI 25-29.9 for non-Asians and 23-26.9 for Asians; Obese = BMI ≥30 for non-Asians and BMI ≥ 27 for Asians

‡ Calculated by taking the median of the highest FIB-4 for each patient

^¶^ Calculated by taking the median of the highest NFS for each patient
